# Induced and Evoked Properties of Vibrotactile Adaptation in the Primary Somatosensory Cortex

**DOI:** 10.1155/2019/5464096

**Published:** 2019-02-20

**Authors:** Nicolaas A. J. Puts, Richard A. E. Edden, Suresh Muthukumaraswamy, Krish D. Singh, David J. McGonigle

**Affiliations:** ^1^Russell H. Morgan Department of Radiology and Radiological Science, The Johns Hopkins University School of Medicine, 600 N Wolfe Street, Baltimore, MD 21287, USA; ^2^F.M. Kirby Research Center for Functional Brain Imaging, Kennedy Krieger Institute, 707 N Broadway Street, Baltimore, MD 21205, USA; ^3^Cardiff University Brain Research Imaging Centre (CUBRIC), School of Psychology, Maindy Road, Cardiff CF24 4HQ, UK; ^4^School of Biosciences, Cardiff University, CF10 3AX Cardiff, UK; ^5^Schools of Pharmacy and Psychology, University of Auckland, Private Bag 92019, Auckland 1142, New Zealand

## Abstract

Prolonged exposure to afferent stimulation (“adaptation”) can cause profound short-term changes in the responsiveness of cortical sensory neurons. While several models have been proposed that link adaptation to single-neuron dynamics, including GABAergic inhibition, the process is currently imperfectly understood at the whole-brain level in humans. Here, we used magnetoencephalography (MEG) to examine the neurophysiological correlates of adaptation within SI in humans. In one condition, a 25 Hz adapting stimulus (5 s) was followed by a 1 s 25 Hz probe (“same”), and in a second condition, the adapting stimulus was followed by a 1 s 180 Hz probe (“different”). We hypothesized that changes in the mu-beta activity band (reflecting GABAergic processing) would be modulated differently between the “same” and “different” probe stimuli. We show that the primary somatosensory (SI) mu-beta response to the “same” probe is significantly reduced (*p* = 0.014) compared to the adapting stimulus, whereas the mu-beta response to the “different” probe is not (*p* = n.s.). This reduction may reflect sharpening of the spatiotemporal pattern of activity after adaptation. The stimulus onset mu-beta response did not differ between a 25 Hz adapting stimulus and a 180 Hz probe, suggesting that the mu-beta response is independent of stimulus frequency. Furthermore, we show a sustained evoked and induced desynchronization for the duration of the adapting stimulus, consistent with invasive studies. Our findings are important in understanding the neurophysiology underlying short-term and stimulus-induced plasticity in the human brain and shows that the brain response to tactile stimulation is altered after only brief stimulation.

## 1. Introduction

Prolonged exposure to afferent stimulation can cause profound changes in the responsiveness of cortical sensory neurons. This process is commonly referred to as “adaptation” and occurs over a number of timescales ranging from milliseconds to minutes (reviewed in [1], see also [2]). While the effects of adaptation at the single-cell level typically lead to a time-dependent decrement of neuronal responsiveness, a number of studies have shown *improvements* in behavioral performance following exposure to an “adaptor”: for example, a long (10–20 s) vibrotactile stimulus improves subsequent vibrotactile frequency discrimination at the same skin site [3]. The size of the effects of adaption on behavior relies upon there being similarities between the stimuli used: for example, a 25 Hz vibrotactile adapting stimulus will produce effects on subsequent vibrotactile amplitude discrimination at 25 Hz, but not at 200 Hz [4]. The duration of the stimulus is also significant, with longer stimuli producing larger adaptation effects.

While a number of models have been proposed that link adaptation to single-neuron dynamics [5–7], this process is currently imperfectly understood at a whole brain level in humans. An important feature of the process is the gradual evolution of adaptation-related activity over the time course of the adapting stimulus. Using optical imaging in nonhuman primates during tactile stimulation, Simons and colleagues revealed that the spatial pattern of activity in the primary somatosensory cortex (SI) evoked by long-duration vibrotactile stimulation [8] follows a highly stereotypical path, with a diffuse initial activation evolving into a more discrete pattern. As links have been made between GABAergic processing and the behavioral effects of adaptation, it has been suggested that the changes in activity within SI represent lateral or feed-forward inhibitory processes: in particular, Tommerdahl and colleagues have suggested that the adaptor allows for a short-term shift in more broadly tuned frequency-specific receptive fields to the adapting frequency, allowing for a net gain in sensitivity when the subsequent stimuli to be discriminated are delivered [7]. Thus, monitoring both the neurophysiological correlates of the adaptor and of the following stimuli is key to understanding how adaptation may produce its behavioral effects. Given the role of adaptation in daily life, as well as its potential impairments in neurodevelopmental disorders such as autism, it is important to gain a better understanding of the neurophysiological correlates underlying adaptation.

However, as the majority of studies mapping SI in humans have used spatiotemporally discrete stimuli to map activity, it is currently unclear if similar processes operate in humans. In this paper, we used magnetoencephalography (MEG) to examine the neurophysiological correlates of adaptation within SI in humans. Utilizing the high temporal resolution of MEG allows for the changes in oscillatory activity over extended periods to be analyzed. We used a 25 Hz adapting stimulus and both a 25 Hz and 180 Hz “probe” stimulus to examine the effects that the period of adaptation has on these two stimuli – one in the flutter range and one in the vibratory range. These two different frequencies were chosen because they are thought to be processed in the same neuronal population within SI (for a review see; [7]). We hypothesize that changes in the mu-beta activity band (15–30 Hz; previously suggested to underlie GABAergic processing within SI; [9]) due to 25 Hz adaptation will be modulated differently for the “same” and “different” probe stimuli.

## 2. Materials and Methods

### 2.1. Participants

12 participants (6 male; mean age 30 yrs., std, 4.8), all right-handed. All procedures were reviewed by the ethics committee of Cardiff University's School of Psychology and conforms with the declaration of Helsinki. All participants gave informed consent.

### 2.2. Tactile Stimulation

**Figure 1:**
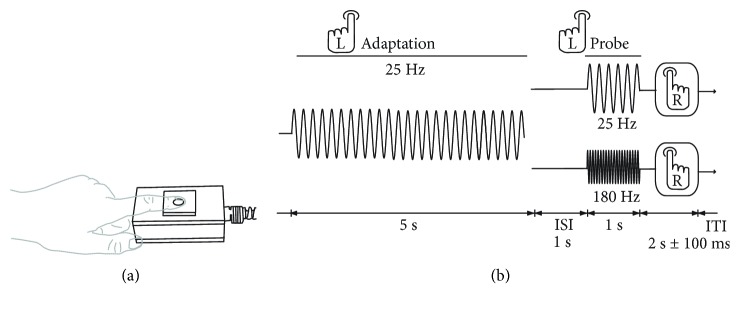
Task as performed during MEG. (a) Visual representation of the left index finger's position on the vibrotactile stimulator. (b) Schematic representation of a single experimental trial. All stimuli were presented to the glabrous skin of distal pad of the participant's left index finger. In both conditions, trials began with the presentation of a 25 Hz “adapting stimulus” (5 s duration), followed by a 1 s gap. The properties of the next stimulus – the “probe” – varied between conditions: in the “same” condition, the stimulus frequency was 25 Hz, but in the “different” condition, it was 180 Hz. Both “probe” stimuli lasted for 1 s. Participants were asked to press a button using their right index finger (R) after each trial to signal their continued attention during the experiment.

Stimulation was delivered using a MEG piezoelectric stimulator [10, 11]. A static surround limited stimulation to the region is directly contacted by the 8 mm diameter tip (the left index finger, LD2). All stimulation was delivered to the glabrous skin of left digit 2 (index finger). Stimuli were delivered via the audio output of a laptop computer (Sony Vaio VGN-NS20M, Realtek high-definition audio) using Matlab 2008b (The MathWorks, 2008). Each trial comprised two separate stimuli: the “adapting stimulus,” a 5 s 25 Hz vibration, and a 1 s “probe” of 25 Hz in condition 1 and 180 Hz in condition 2 (Figure 1). A 1 s gap separated the two stimuli. Each condition was acquired separately and consisted of 100 trials (ITI 2 s ± 100 ms). The amplitude for the 25 Hz stimuli was set at a suprathreshold value, and the amplitude of the 180 Hz stimulus was set at 10% of the 25 Hz amplitude to control for differences in subjective magnitude (see also [12]). During the experiment, participants fixated on a small cross on a Mitsubishi Diamond Pro 2070 monitor controlled by Matlab software (1024 × 768 resolution, 100 Hz refresh rate). Participants were asked to press a button on a LUMItouch response box (LUMItouch, Photon Control Inc., Burnaby, Canada), using their right index finger as soon as the “probe” stimulus finished, to maintain attention. Prior to the task, participants received a practice session in which they were exposed to exemplar vibrotactile stimuli of various frequencies and amplitudes in both the flutter and vibration range. Once participants reported they were comfortable with discriminating the different stimuli, they received a practice session of the passive adaptation task. The practice session consisted of ten trials consisting of a 5-second stimulus, followed by a single probe. The order of conditions was randomized between participants.

### 2.3. MEG Acquisition and Analysis

Data were acquired continuously using a whole-head CTF 275 channel MEG radial gradiometer system sampled at 1200 Hz (0–300 Hz band pass). Two of the 275 channels were turned off due to excessive sensor noise. An additional 29 reference channels were recorded for noise cancellation purposes, and the primary sensors were analyzed as synthetic third-order gradiometers [13]. Prior to data analysis, trials with obvious artefacts such as head movements, eye blinks, and muscle activity were excluded from further analysis. Two separate datasets were generated, one based on the adapting stimulus (−1 to 7 s) and one on the basis of the probe stimulus (−1 to 2 s with probe onset at zero).

For each participant, a 1 mm^3^ isotropic-resolution T1-weighted anatomical scan (FSPGR) was acquired. For source localization, a multiple-local-spheres forward model [14] was derived by fitting spheres to the brain surface (one sphere for each sensor) extracted by the FSL Brain Extraction Tool [15]. To facilitate the localization of SI, we utilized a synthetic aperture magnetometry event-related field (SAMerf [16]) approach: the computed evoked response was filtered between 0 and 90 Hz beamformer weights to create three-dimensional SAMerf images of source power (pseudo-*t* statistics) for 1 second of baseline (−1–0 seconds) compared to 10 ms bins spanning between 0 and 150 ms post-stimulus for each participant. SAM images used to manually detect peak location in SI (expected between 60–70 ms) for each participant and the location of the peak activity was confirmed using the anatomical MRI of the individual.

**Figure 2:**
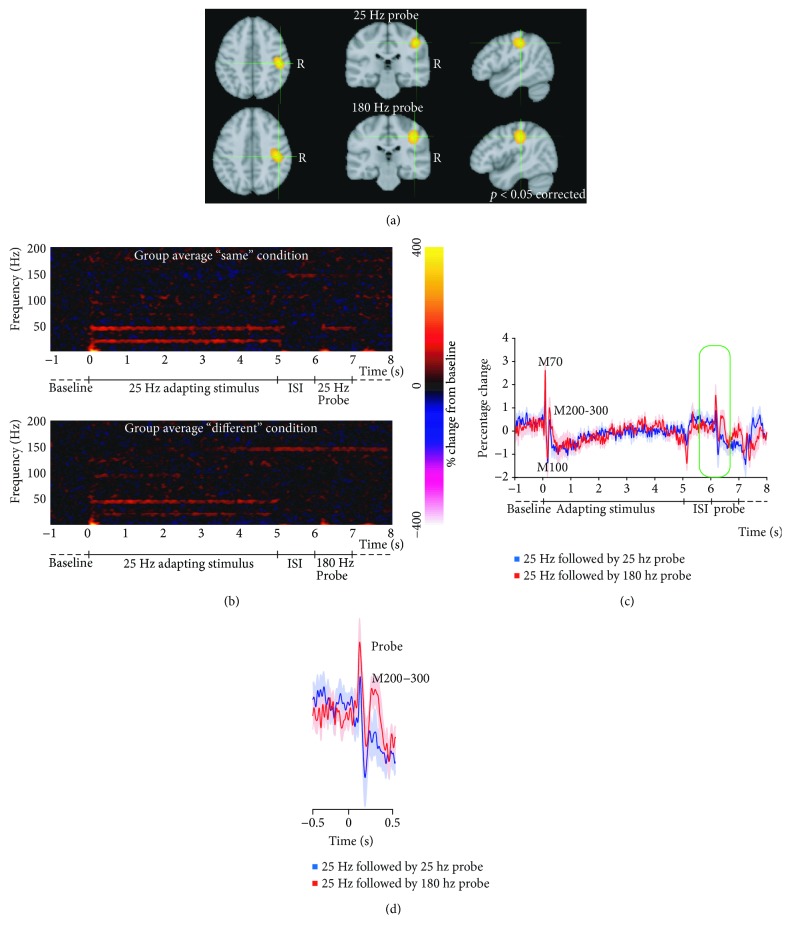
Group MEG results demonstrating differences between responses to “same” (25 Hz) and “different” (180 Hz) probes. (a) Statistical maps showing significant group activation clusters for the 25 Hz and 180 Hz probe stimuli, displayed on the MNI-152 template brain. Maps are thresholded at *p* < 0.05 (thresholded using nonparametric permutation testing for the omnibus statistic). Activation can be seen centred on the right somatosensory cortex in both conditions. The locations of the most significant (peak) voxel did not differ significantly between the 25 Hz and 180 Hz probe. (b) Group average evoked activity filtered between 0 and 200 Hz for the “same” (top) and “different” (bottom) condition. Power is expressed as percentage change compared to baseline (−1–0 seconds before the adapting stimulus onset). Both panels show a characteristic steady-state response (SSR) at 25 Hz for the adapting stimulus with putative harmonics at 50 Hz. A 25 Hz SSR is shown for the 25 Hz probe in the top panel, but a 180 Hz SSR cannot be distinguished for the 180 Hz probe in the bottom panel. (c) The trace between 0 and 20 Hz (to omit SSR effects) is not significantly different for the adapting stimulus between the two conditions and shows characteristic M70, M100, and M200-M300 peaks. (d) The M200-M300 for the 180 Hz probe appears stronger than for the 25 Hz probe, but this is not significant (*p* = 0.068).

A group SAM analysis was performed to contrast the first peak of SI activity between the flutter (25 Hz) and vibratory (180 Hz) stimuli, between the active periods (30–100 ms after stimulus onset) of the probe in both conditions. For the production of grand-average SAM maps, individual SAM images were first spatially normalized onto the MNI (T1) average brain using FLIRT [17]. Nonparametric permutation testing for statistical significance of the group peak SI activity was performed using 4096 permutations for each condition separately, and thresholded using the omnibus test statistic at *p* < 0.05 [18, 19]. To compare conditions, a voxel-wise *t*-statistic image was then calculated using the intersubject variability and a nonparametric permutation performed, using 2^*n*^ (*n* = number of subjects) permutations. This created an estimate of the *t*-value distribution for the null hypothesis, which was that the 25 Hz and 180 Hz probe stimuli evoke identical voxel-wise activity. The images presented in Figure 2(a) are the grand average across all participants. To account for multiple comparisons, the largest *t*-value in each permutation was used to create a probability map for the range of largest *t*-values and only unpermuted *t*-values larger than this threshold were significant (at *p* < 0.05) [18, 19].

To reveal time-frequency responses at these locations, “virtual sensor” recordings were generated at the individual peak locations using the Hilbert transform and then averaged across subjects, yielding percentage changes in neuromagnetic activity from the average baseline value for both evoked and induced MEG responses. Virtual sensors were band-pass filtered between 0 and 200 Hz at 0.5 Hz frequency step intervals, using an 8 Hz wide band pass, third-order Butterworth filter. For the evoked (phase-locked) activity, the amplitude envelope and time-frequency data was obtained using the Hilbert transform between 1 and 200 Hz in 0.5 Hz frequency steps, expressed as the percentage change from prestimulus baseline (−1 to 0 s). This evoked activity was averaged across participants for each condition. For further investigation of power changes across time, evoked activity was plotted between 0 and 20 Hz for the duration of the trial to exclude the effect of higher frequency stimulus-induced steady state responses (SSRs).

Using ethologically valid stimuli such as vibrotactile stimulation produces neuromagnetic activity in SI with a longer latency than direct electrical stimulation of nerve fibers. The first response seen within the epoched data occurs around 70 ms (M70), with subsequent peaks occurring at 150 ms (M150) and between 200 and 300 ms (M200-300) post-stimulus onset [20–24]. To look at specific frequency bands in the induced (non-phase-locked) activity, time-frequency analysis was performed on both adapting stimulus and probe separately. Peak mu-alpha (7-15 Hz) and mu-beta (15-30 Hz) band frequency and amplitudes, expressed as percentage change from baseline, were extracted for the induced activity. Differences between conditions (adapting stimulus, 25 Hz probe, and 180 Hz probe) were measured as significant differences in power for the mu-alpha and mu-beta separately as measured from 100-1000 ms after stimulus onset (for the typical event-related desynchronization (ERD) and synchronization (ERS)) and analyzed using one-way ANOVA with Bonferroni correction for multiple individual comparisons. Finally, peak mu-band frequency (7-30 Hz) was extracted for the probe duration and compared against mu-band frequency for the adapting stimulus (one-way ANOVA with Bonferroni correction).

## 3. Results

One participant was excluded due to excessive motion. The 25 Hz and 180 Hz probe stimuli both produced clear peaks within SI in the group SAM analysis (XYZ; 25 Hz; 48.2-28.1-44; 180 Hz; 44.2-28.1-44), but *t*-weighted comparison analysis between the two group SAM images did not show significant differences between the two locations (independent locations shown in Figure 2(a) at *p* < 0.05 using nonparametric permutation testing for statistical significance of the group peak SI activity thresholded using the omnibus test statistic at *p* < 0.05).

The average evoked activity between 0 and 200 Hz was plotted for the entire duration of the trial (Figure 2(b)). In the evoked response for the adapting stimulus and probes, significant increases in power mirroring the stimulation frequency were seen. The top panel of Figure 2(b) shows a characteristic SSR at 25 Hz with putative harmonics at 50 Hz for the adapting stimulus and 25 Hz probe. In the bottom panel, no SSR at 180 Hz is visible, possibly due to different absolute amplitudes in the cortical representation for vibration. Analysis of the mean-corrected evoked response between 0 and 20 Hz (to exclude an effect of the SSR) shows a similar trace for the adapting stimulus in both conditions (see Figure 2(c), no significant differences). A characteristic positive deflection (M70) is followed by a negative deflection (M100) followed by an upward positive deflection (M200-M300) for both adapting stimuli and probes (see [25, 26]). Activity for M200-M300 for the probe (see Figure 2(c)) shows a stronger component for the 180 Hz probe than for the 25 Hz probe, but while there is a trend for a difference, this is not significant (two-sample paired *T*-test of evoked power between 200 and 600 ms after onset of the probes, *p* = 0.068).

**Figure 3:**
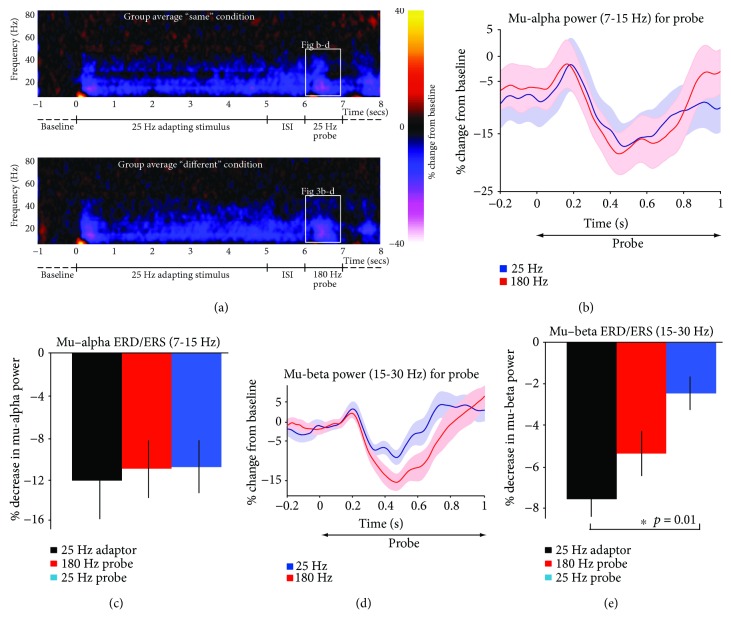
Analysis of induced responses. (a) Time frequency plot of induced group average activity between 0 and 80 Hz for the “same” condition (top panel) and the “different” condition (bottom panel. Shown is percentage change from baseline (−1 to 0 ms before adaptor stimulus onset) as shown in the color bar. As can be seen in both plots, an initial desynchronization in the mu-beta band (15–30 Hz) is followed by a small resynchronization but activity remains desynchronized for the duration of the adapting stimulus as well as the ISI. The white boxes outline the data shown in (b) and (d). (b) Average power envelope across the mu-alpha band (7-15 Hz), reported as a change compared to baseline, for the probe conditions. (c) There were no differences in mu-alpha power between the 25 Hz (blue) and 180 Hz (red) probe. (d) Average power envelope across the mu-beta band (15-30 Hz), reported as a change compared to baseline, for the probe conditions. (e) Average mu-beta power across the probe duration was significantly reduced for the 25 Hz probe (“same” condition, blue) compared to the adapting stimulus (black), but not for the 180 Hz probe (in red; “different” condition) compared to the adapting stimulus (black).

The induced response shows a sustained reduction in power for the mu-alpha and mu-beta bands for the duration of the adapting stimulus in both conditions (Figure 3(a)) compared to the baseline period. No differences in power were found between conditions for the two (identical) adapting stimuli for the duration of the stimulus. No significant differences were found between the 25 and 180 Hz probes in the mu-alpha range (Figures 3(b) and 3(c)), but the mu-beta response shows a significant reduction for the 25 Hz probe when compared to the adapting stimulus (Figures 3(d) and 3(e)). The amplitude of the mu-beta response was significantly different from the adapting stimulus for the 180 Hz probe (one-way ANOVA) and showed a significant difference between conditions (df = 2, *F* = 4.928, *p* = 0.014). Further post hoc analysis with Bonferroni correction shows that the magnitude of the mu-beta ERD/ERS (beta power envelope between 100 and 1000 ms after stimulus onset) is significantly smaller for the 25 Hz probe than for the 25 Hz adapting stimulus (*p* = 0.012), whereas no significant difference was found between the 25 Hz adapting stimulus and the 180 Hz probe (*p* > 0.5) as shown in Figure 3(e). There was no significant difference between the mu-beta power between the two probes although a trend can be seen. Analysis of mu-*frequency* between the 25 Hz and 180 Hz conditions shows that the average mu-frequency for the ERD/ERS complex is significantly lower for the 25 Hz probe than for the 180 Hz probe (*p* = 0.044) but not compared to the adapting stimulus. The 180 Hz probe was also not significantly different from the adapting stimulus (*p* > 0.2).

## 4. Discussion

Our results show that the mu-beta power is significantly different when the adapting stimulus and subsequent probe are the same, but not when they were different. It is possible that the behavioral improvement seen in this kind of adaptation protocol [3] is reflected by changes in the spatiotemporal pattern of activity of subsequent stimuli reflected in the mu-beta band.

Group analyses showed no significant difference in peak location between the 25 Hz and 180 Hz stimuli. This is consistent with invasive studies [27, 28] showing that the same neuronal population within SI becomes activated after stimulation with either 25 Hz or 200 Hz.

An SSR at 25 Hz and its harmonics were visible for the 25 Hz adapting stimulus and 25 Hz probe, showing a neuromagnetic response at the vibrotactile stimulus' driving frequency that continues for the duration of the stimulus. However, we were not able to see an SSR at 180 Hz. Tommerdahl et al. [27, 28] have shown that vibration leads to a weaker optical imaging response in SI compared to flutter (our 25 Hz stimulus). As we adjusted the stimulus amplitude on the vibratory stimulus (higher frequencies feel stronger), this may be responsible for the lack of a 180 Hz SSR. It is also possible that timing inaccuracies affect the phases of higher frequencies more, and therefore, jittering results in a smaller 180 Hz stimulus signal, not visible in our analysis.

The evoked activity in SI for both flutter and vibration shows the expected peaks at the M70 and M100 latencies. Both conditions also show a typical M200-300 component for the adapting stimulus and the 180 Hz probe, but may be reduced for the 25 Hz probe, potentially reflecting a larger “memory” component when the adapting stimulus and probe are different. The difference is not likely to be driven by the difference in stimulus frequency which could have differential impact on the neuronal population. Different, or larger, population responses would have likely been reflected in the early evoked response as well as these early responses more readily reflect local processing, but we find no differences in these early responses. We believe this finding likely reflects feedback from higher cortical areas rather than local processing [26]. Repetitive stimulation may also lead to adaptation in higher cognitive regions that is then fed back to SI.

A 5 s stimulus is longer than typically used in tactile experiments. For our 5 s duration adapting stimulus, we found that activity in SI is reflected by an ERD followed by a small ERS, followed by a *sustained* desynchronization compared to baseline, for the duration of the adapting stimulus (as seen in Figure 3). Thus, the adaptor seems to engender a change in endogenous SI dynamics: while the initial ERD/ERS occurs at the start of stimulation, the adapting stimulus remains in desynchronized “state” throughout its presentation, which may reflect its “dampening” effect on subsequent stimulation.

Our results show a significant difference in mu-beta power between the “same” and “different” conditions. The mu-beta ERD/ERS between 100 and 1000 ms after probe-stimulus onset is weaker than the mu-beta ERD/ERS for the adapting stimulus when the probe is the same frequency as the adapting stimulus but not when the probe is *different* from the adapting stimulus. These results show that while a 25 Hz adapting stimulus and a 180 Hz probe stimulus show equivalent mu-beta ERD/ERS responses (despite differing in frequency and stimulus-duration), a 25 Hz probe preceded by a 25 Hz adapting stimulus has a weaker mu-beta ERD/ERS, suggesting that its spatiotemporal pattern of activity is modulated by the prior adapting stimulus, or at least to a stronger degree than when the probe stimulus is different. This is unlikely to occur due to the probe being shorter than the adapting stimulus. If anything, the ERD/ERS would be expected to be smaller for the adapting stimulus, consistent with optical imaging studies showing a reduced and more discrete area of activity for long-duration stimulation. Further, these results show that the mu-beta response to tactile stimulation is the same irrespective of whether vibrotactile stimulation is delivered in the flutter or vibration ranges. In addition, the results confirm that power in the mu-beta band does not directly reflect the stimulus characteristics of afferent stimulation, but may be indicative of an integration of prior activity with afferent input.

These results show that the network state within SI affects subsequent processing of the “probe” in the mu-beta band range but not in the mu-alpha range. These indicate the functional role of the beta rhythm in shaping the response of SI to afferent input, whereas the mu-alpha rhythm is more concerned with suppression of inactivation during a task, irrespective of stimulus characteristics. It remains unclear what underlying neurobiological mechanism drives the differences in mu-beta ERD/ERS. Previous studies have suggested a role of the mu-beta rhythm in discrimination [29, 30], but while not excluding the suggestion that mu-beta is involved in discrimination, we show more specifically that the mu-beta rhythm is affected by subsequent stimulation in a nondiscrimination task prior to decision making and discriminatory aspects of sensory processing.

It is likely that GABAergic inhibition plays a role in these changes. Invasive studies have shown that the efficacy of excitatory pyramidal cells is reduced, but the efficacy of inhibitory interneurons increases [1, 31] as a result of adaptation and the “relative strength of excitation and inhibition in a cortical circuit would be expected to change” [1]. As repetitive stimulation has been shown to cause increased activity in the stimulated region but progressive decreases in activity in neighboring regions, inhibitory mechanisms may play a role. Juliano et al. [32] showed that application of the GABA antagonist bicuculline leads to a more diffuse cortical response to repetitive stimulation. In addition, studies investigating autism have shown that the behavioral effect of adaptation is not present in participants with autism [33, 34]. Tommerdahl et al. [33] and Tannan et al. [35] discuss that a deficit in GABA-mediated neurotransmission, particularly local inhibition between cortical minicolumns, underlies this behavioral effect. In addition, Folger et al. [36] investigated the role of NMDA (excitatory) processes on inhibition and showed that adaptation in healthy participants is impaired when NMDA is blocked by dextromethorphan. In addition, mu-beta band oscillations have been associated with the GABAergic inhibitory network in SI [9, 37–39] showing that more GABA is correlated with a larger increase in ERS power in the mu-beta range, and pharmacological increase of GABA leads to increases in motor ERD [40]. Increased activity of GABAergic neurons may underlie adaptation effects, and it would be expected that the mu-beta rhythm would increase as GABAergic activity increases: indeed, we find a smaller desynchronization (i.e., more synchronization) of the mu-beta rhythm after adaptation. An increase in GABAergic activity might aid in tuning the neuronal response to 25 Hz and inhibition of irrelevant information and thus lead to a shift in activity. Furthermore, [41] showed, using optical imaging, that the neuronal response to a subsequent adapting stimulus after the same adapting stimulus was reduced, whereas the response to a novel stimulus was not, similar to our presented data. It is more likely that our data reflect a general “sharpening” in activity due to adaptation controlled by inhibitory interneurons.

A significant limitation of this study is that we did not investigate the effect of a 180 Hz adapting stimulus on a subsequent 25 Hz and 180 Hz probe. This crossover was consciously not applied in this study. The neurophysiological effects of repetitive stimulation in the vibration range (50–180 Hz) are unknown. The behavioral implications of repetitive vibration stimulation are different from flutter stimulation; repetitive stimulation with a vibration stimulus appears to lead to numbing and potentially painful percepts. While an interesting target for future studies, these different mechanisms are beyond the framework of the role of adaptation as addressed in the current study. Given that the physical location of activation for the 25 Hz and 180 Hz probe do not differ and that the mu-beta response is the same for the 25 Hz adapting stimulus and 180 Hz probe, it seems likely that it is the 25 Hz probe which is processed differently. Furthermore, for the 180 Hz stimulus, we used an intensity of 10% of the 25 Hz stimulus. It has been well established that vibratory stimulation is perceived more strongly than flutter stimulation, and the detection threshold is known to be much lower for vibration stimulation. It is possible that the difference in intensity affected our results. However, if this were the case, we would have expected to see reduced evoked activity for the 180 Hz probe and perhaps a smaller desynchronization in the induced response. However, we show that responses are the same for the 25 Hz adaptor and 180 Hz probe, with the response for the 25 Hz stimulus being different, suggesting that stimulus amplitude alone cannot account for our findings.

## 5. Conclusion

In summary, we have measured the neurophysiological response to tactile adaptation, a form of short-term plasticity, for the first time with MEG. Our results show that an adapting stimulus leads to sustained responses in both the evoked and induced activation of SI and that a 25 Hz adapting stimulus differentially affects a “same” 25 Hz probe compared to a “different” 180 Hz probe. These findings are important in understanding the neurophysiology underlying short-term and stimulus-induced plasticity in the human brain and show that the brain response to tactile stimulation is altered after only brief stimulation [2]. These findings may be important for future studies investigating disorders where sensory processing (e.g., adaptation) is impaired, such as autism.

## Data Availability

The processed data used to support the findings of this study are available from the corresponding author upon request.

## Abbreviations

MEG: Magnetoencephalography
SI: Primary somatosensory cortex
SAM: Synthetic aperture Magnetometry
ERF: Event-related field
SSR: Steady-state response
ERD: Event-related desynchronization
ERS: Event-related synchronization.
